# Malaria risk factors and care-seeking behaviour within the private sector among high-risk populations in Vietnam: a qualitative study

**DOI:** 10.1186/s12936-017-2060-0

**Published:** 2017-10-16

**Authors:** Ingrid Chen, Huong Ngo Thi Thanh, Andrew Lover, Phung Thi Thao, Tang Viet Luu, Hoang Nghia Thang, Ngo Duc Thang, Josselyn Neukom, Adam Bennett

**Affiliations:** 1 Malaria Elimination Initiative, Global Health Group, University of California, San Francisco, 550 16th Street, 3rd Floor, San Francisco, CA 94158 USA; 2 Population Services International Vietnam, VinaFor Building, 127 Lò Đúc, Đồng Xuân, Hanoi, Vietnam; 3 National Institute of Malaria, Parasitology, and Entomology (NIMPE), Vietnam, 35 Trung Van, Tu Liem, Hanoi, Vietnam

**Keywords:** Vietnam, Malaria, Care-seeking behavior, Private sector, High-risk population, Migrant and mobile populations

## Abstract

**Background:**

Vietnam has successfully reduced malaria incidence by more than 90% over the past 10 years, and is now preparing for malaria elimination. However, the remaining malaria burden resides in individuals that are hardest to reach, in highly remote areas, where many malaria cases are treated through the informal private sector and are not reported to public health systems. This qualitative study aimed to contextualize and characterize the role of private providers, care-seeking behaviour of individuals at high risk of malaria, as well as risk factors that should be addressed through malaria elimination programmes in Vietnam.

**Methods:**

Semi-structured qualitative interviews were conducted with 11 key informants in Hanoi, 30 providers, 9 potential patients, and 11 individuals at risk of malaria in Binh Phuoc and Kon Tum provinces. Audio recorded interviews were transcribed and uploaded to Atlas TI™, themes were identified, from which programmatic implications and recommendations were synthesized.

**Results:**

Qualitative interviews revealed that efforts for malaria elimination in Vietnam should concentrate on reaching highest-risk populations in remote areas as well their care providers, in particular private pharmacies, private clinics, and grocery stores. Among these private providers, diagnosis is currently based on symptoms, leaving unconfirmed cases that are not reported to public health surveillance systems. Among at-risk individuals, knowledge of malaria was limited, and individuals reported not taking full courses of treatment, a practice that threatens selection for drug resistance. Access to insecticide-treated hammock nets, a potentially important preventive measure for settings with outdoor biting Anopheles vectors, was also limited.

**Conclusions:**

Malaria elimination efforts in Vietnam can be accelerated by targeting improved treatment, diagnosis, and reporting practices to private pharmacies, private clinics, and grocery stores. Programmes should also seek to increase awareness and understanding of malaria among at-risk populations, in particular the importance of using preventive measures and adhering to complete courses of anti-malarial medicines.

**Electronic supplementary material:**

The online version of this article (doi:10.1186/s12936-017-2060-0) contains supplementary material, which is available to authorized users.

## Background

Over the past 15 years, malaria mortality has dropped by approximately 62% globally, fueling ambitions to eradicate malaria by 2040 [1, 2]. As malaria endemicity declines, countries preparing for malaria elimination strive to identify, treat, and document all cases [3, 4]. Often, the remaining cases during elimination stages tend to be clustered in certain ecologies, where risk of ongoing transmission is contingent on human behaviour patterns and select groups of individuals have higher risks of infection [5, 6].

In Southeast Asia, where anopheline vectors often preferentially feed on human hosts in outdoor environments, malaria ecology presents multiple challenges [7]. Not only do outdoor biters such as *Anopheles minimus*, *Anopheles epiroticus*, and *Anopheles dirus* vector complexes need to be confronted in this region, but this must be done rapidly, as Southeast Asia is the nucleus of *Plasmodium falciparum* drug resistance against the first-line artemisinin-class drugs, a global threat that has emerged repeatedly in the region since 2006 [8–10].

Against this backdrop, Vietnam, where the malaria caseload has been effectively reduced by 93% between 2000 and 2015 (from 274,910 down to 19,252 cases), is currently confronting challenges to malaria elimination [11]. Malaria in Vietnam is concentrated in rural, forested areas, particularly in the central highlands and areas bordering southern Lao PDR and southeastern Cambodia. Moreover, it is a disease of the rural poor, with the highest risk of infection presiding among migrant workers and ethnic minorities [12–14]. Drug resistance against both dihydroartemisinin and piperaquine, the two components of the recommended first-line treatment for *P. falciparum* malaria, has been reported in-country [15, 16]. To counter artemisinin resistance, Vietnam has issued a ban on oral artemisinin monotherapies (Decision No 4718/QD-BYT issued by MOH in 2014).

In order to achieve malaria elimination in Vietnam, malaria cases among at-risk populations must be detected, treated and documented. However, these individuals are believed to seek malaria treatment through informal, private healthcare providers, a poorly described part of the health sector escaping the reach of the many interventions delivered through the public sector [1, 17]. There is no formal documentation on how often the private sector is used in Vietnam for malaria care, which types of private providers are accessed, nor the types of clientele that seek care through this sector [18]. This qualitative study investigates the current composition and role of private providers in malaria care-seeking behaviour, as well as the context and characteristics of their clientele, to inform malaria programme design in Vietnam.

## Methods

### Study overview

This study was comprised of two components: (1) Semi-structured key informant interviews were conducted with individuals working on malaria control efforts in Vietnam and/or with the informal private sector, in order to gain expert opinion and insight on the composition and role of the private sector in rural areas nationwide, in particular those that are endemic with malaria (January to March, 2016); (2) semi-structured interviews were conducted with private providers and patient/at-risk individuals in remote malaria-endemic locales, to contextualize and characterize providers and their clientele (December 2015 to January 2016). For providers, this included an exploration of what they were selling, what their operating practices were, and how they communicated, to establish potential methods they could use to report malaria cases currently missed by the public sector surveillance system. Clientele selection focused on those at risk of malaria, seeking to establish high risk behaviours threatening malaria elimination efforts that need to be addressed, as well as contextual factors such that interventions can be designed to maximize adherence from individuals at risk. In areas selected for field study, the highest malaria transmission occurs during the rainy season, from April through November, and high-risk individuals are usually workers in forests and farms, many of whom are migrants [19, 20].

### Sampling and locations

#### Key informant interviews

Key informant interviews were conducted in Hanoi, where many non-governmental organizations (NGOs), the Ministry of Health, and other healthcare organizations are located. Key informants were selected using purposive sampling through networks of programme and organizational staff members (through the University of California, San Francisco (UCSF) Global Health Sciences department and Population Services International (PSI) Vietnam), and any colleagues suggested by members of those networks, to identify approximately ten key informants.

#### Provider, patient and at-risk individual interviews

Preliminary feasibility assessments for study sites were conducted in three provinces in the central highlands, where malaria endemicity is highest in Vietnam. All provinces investigated also have the presence of artemisinin-resistant *P. falciparum* parasites, thus taking place where malaria elimination efforts are most urgently needed. All areas assessed were remote, rural and mountainous malaria endemic areas with poor infrastructure and living conditions.

In each district, researchers sought to establish the density of private providers involved in malaria treatment and/or diagnostic practices. This included speaking with village leaders and local authorities, and asking village leaders, motorcycle drivers, tea shop and restaurant owners, and/or small grocery shop operators to identify patients and providers, who were then sought using a variety of interpersonal approaches to build rapport. The interview questionnaires were also piloted in these locations, and revised following insights gained.

**Fig. 1:**
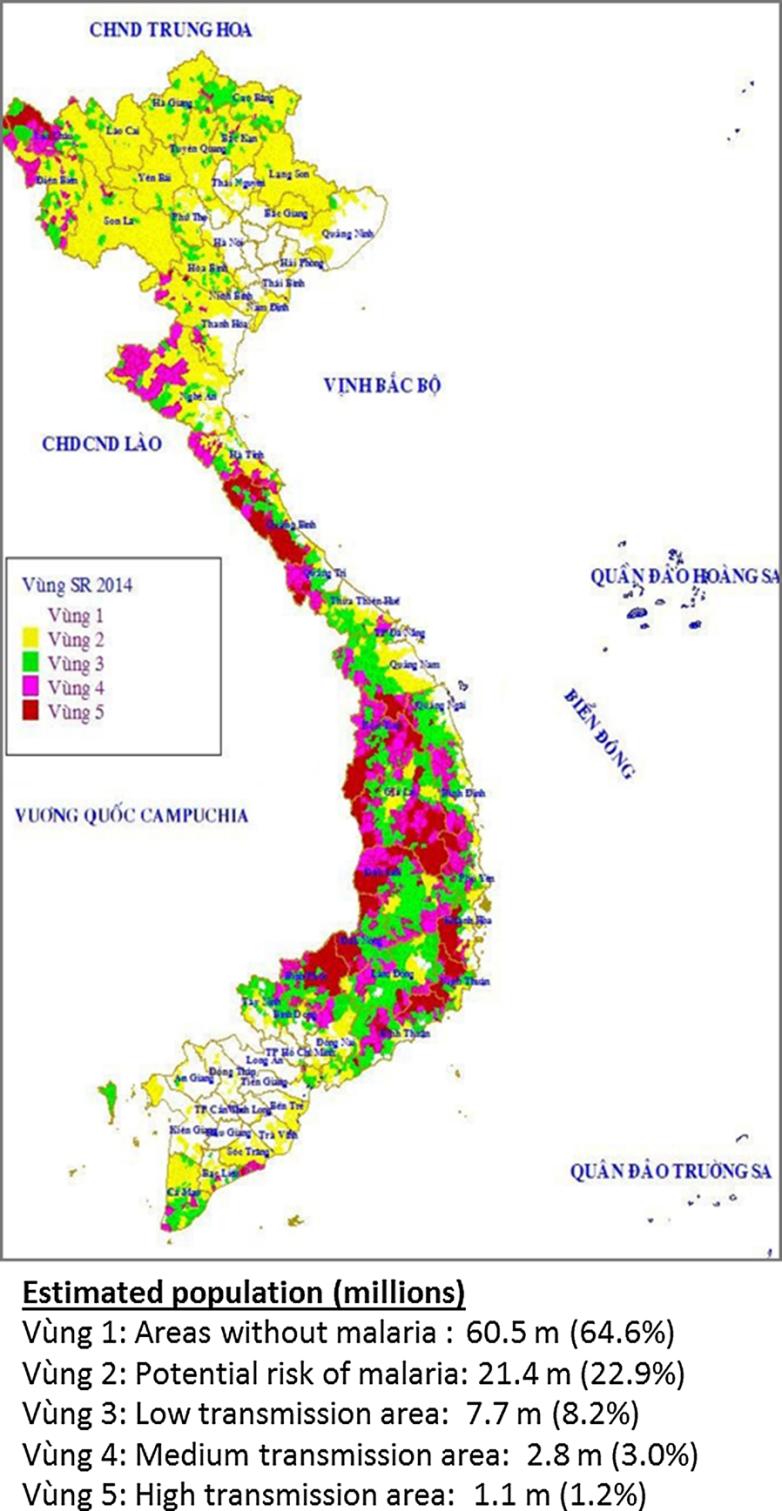
Malaria risk stratification map, 2014

**Fig. 2:**
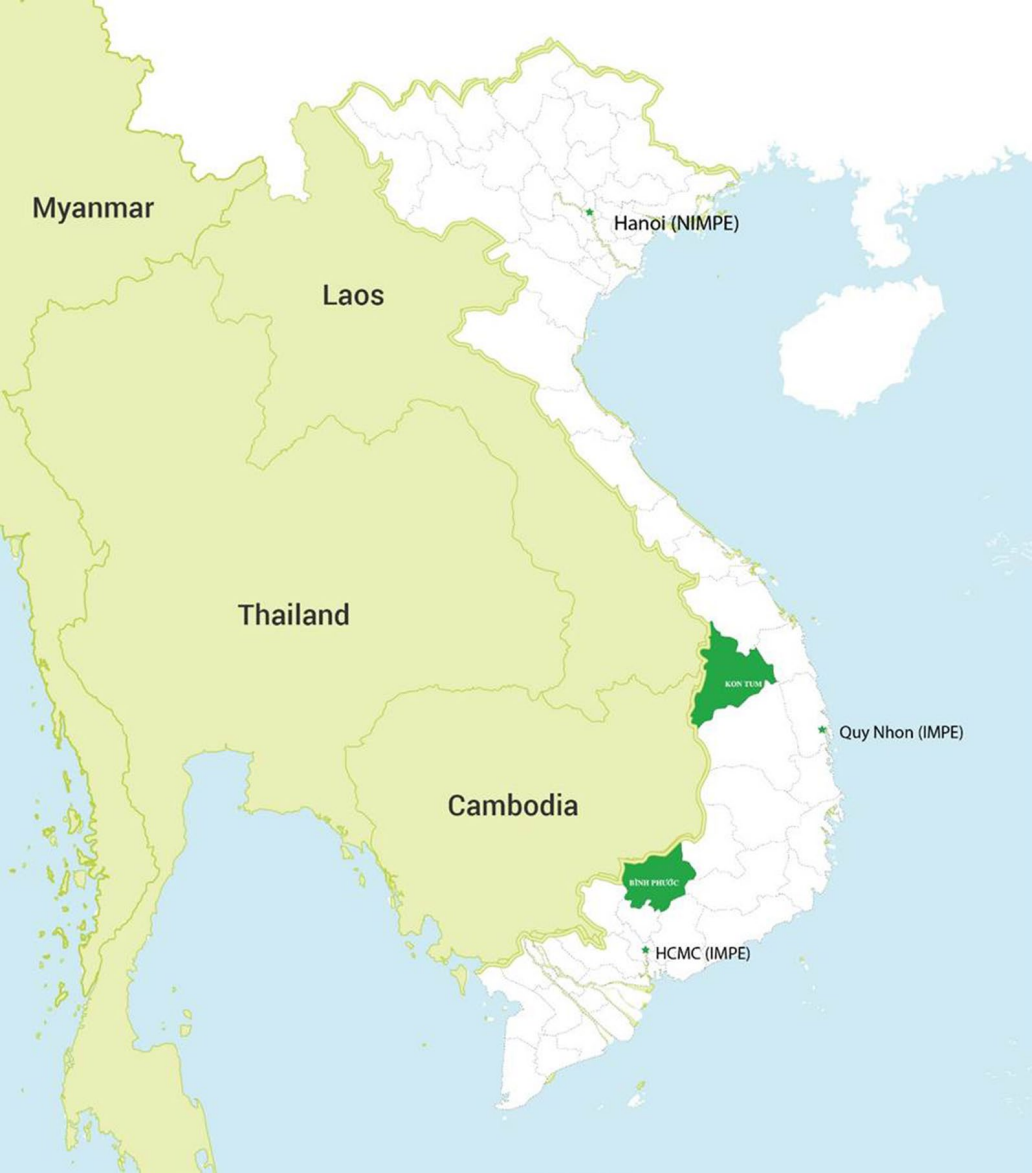
Provinces of field study, qualitative studies December 2015–January 2016, Vietnam

The final selection of provinces was based on a combination of high malaria endemicity, higher numbers of private providers found, as well as the political feasibility of acquiring permits to conduct research in each area (Fig. 1; Table 1). Areas selected for study included Binh Phuoc, located in Southwestern Vietnam, was known to be a province with high malaria endemicity and the presence of artemisinin-resistant parasites (Tier 1), and Kon Tum, where artemisinin-resistant *P. falciparum* malaria (Tier 2) was documented. Kon Tum also contains porous borders with both Laos and Cambodia thought to be crossed by migrant workers, posing a risk of malaria importation from neighbouring countries (Fig. 2) [21].

**Table 1:** Malaria epidemiology in provinces selected for feasibility assessments

Province	Percentage of individuals at risk for malaria (%)	Deaths attributed to malaria	Confirmed cases			
			All malaria infections	P.f	P.v.	Mixed infections
Quảng Nam	0.66	0	369	125	242	2
Kon Tum	1.06	0	179	79	99	1
Đắk Lắk	0.53	1	779	347	424	8
Bình Phước	1.92	1	1799	1000	706	93

P.f., *Plasmodium falciparum*; *P. v., Plasmodium vivax* malaria

In Binh Phuoc, which had a total population of approximately one million individuals, there were 1799 confirmed cases of malaria in 2016 (one death attributed to malaria), 56% were *P. falciparum* infections, 39% were *Plasmodium vivax* infections, and the remaining 5% were mixed infections. Binh Phuoc is a predominantly rural province, with only 15% of the population living in towns. This province is divided into 11 district-level subdivisions. The majority of individuals in Binh Phuoc are Vietnamese, although ethnic minorities are present throughout the province, comprising of Xtiêng, Nùng, Tày, and Khmer minorities. Binh Phuoc is one of the most agriculturally productive provinces of Vietnam, whose economy heavily relies on cash crops such as cashew nuts and rubber.

In Kon Tum, which had a population of approximately 330,000 in 2016, there were 179 confirmed malaria cases (zero deaths attributed to malaria); 44% were *P. falciparum* infections, 55% were *P. vivax* infections, and the remaining 1% were mixed infections. Kon Tum also has a primarily agricultural economy, and is one of the least populated provinces in Vietnam. The capital, Kon Tum, is its own municipality. There are 76 communes, 10 wards, and 6 towns. Ethnic minorities make up 51% population of Kon Tum, and are mostly comprised of the Ba Na, Xo Dang, Gie Trieng, Gia Rai, B’Rau, and Ro Man minorities, many of whom work on fields in the mountains.

### Recruitment of providers, patients, and at-risk individuals

In each province, Binh Phuoc and Kon Tum, 15 providers and 10 patients or individuals at risk of malaria were interviewed. These participants were identified through conversations with local contacts including village leaders, motorbike taxi drivers, grocery store workers, and mobile vendors. Patients and at-risk individuals, once identified, also helped to refer the study team to providers, and vice versa. In some cases, providers were identified when at-risk individuals used a ‘mystery client’ approach, presenting at private outlets and reporting to have malaria symptoms. If anti-malarial medications and/or malaria rapid diagnostic tests (RDTs) were found on site through this approach, the study team would return to these outlets, approaching providers for interviews. The study team also visited community health stations to search for documented lists of malaria patients, but no cases were reported through this approach.

### Inclusion criteria

#### Key informants

Inclusion criteria for key informant interviews required that each individual interviewed have experience working on malaria control activities and/or engaging informal private providers in Vietnam. All interviews were conducted in the English or Vietnamese language.

#### Private providers

All providers interviewed were either owners or workers in the shop that reported to have anti-malarial drugs in stock within the past 3 months prior to the day of the interview. Additionally, providers were required to be ≥ 18 years of age, and were fluent speakers of the Vietnamese language and/or minority languages.

#### Patients and at-risk individuals

All patients interviewed reported to have either sought healthcare from a private provider for themselves or for a family member (within a year from the date of interview) and were diagnosed with a malaria infection according to public and/or private providers within the last year. All at-risk individuals of malaria reported to have stayed in the forest or on a farm or plantation for at least one night in the last 3 months, a practice that researchers anticipated would identify farmers and forest workers, many of whom are migrant workers. All potential patients and at-risk individuals interviewed were ≥ 18 years of age, and were fluent speakers of the Vietnamese language and/or minority languages.

### Data collection

#### Key informant interviews

A total of 11 key informant interviews were conducted; six key informant interviews were conducted in English (IC), and five interviews were conducted in Vietnamese (HNTT). A semi-structured interview guide was used, and prompts were used to guide the interview (English version shown in Additional file 1. Semistructured Interview guide: key informant). All interviews took place in a private location, were between 45 and 90 min in duration, and were audio recorded and accompanied by hand-written notes. All key informants interviewed agreed to be audio recorded.

#### Interviews with providers, patients, and at-risk individuals

A total of 30 interviews with providers, 9 interviews with patients, and 11 interviews with at-risk individuals were conducted in Binh Phuoc (border districts: Loc Ninh, Bu Gia Map, and Bu Dop; and non-border district Bu Dang) and Kon Tum (border districts: Sa Thay, Ngoc Hoi, Dak Glei; and non-border districts Dak Re and Kon Tum). All interviews were conducted using a semi-structured interview guide (English version of interview guides shown in: Additional file 2. Semistructured Interview Guide: private provider; Additional file 3. Semistructured Interview Guide: malaria patient; and Additional file 4. Semistructured Interview Guide: individual at risk of malaria). Interviews lasted between 45 and 90 min, and were conducted by a researcher (PTT in Binh Phuoc, HNNT in Kon Tum) with a note-taker present. Interviews with providers were conducted in shops, and interviews with patients and at-risk individuals were conducted in locations easily accessible to interviewees, including tea or coffee shops, or in their homes. All interviewees agreed to be audio recorded with the exception of one provider from Binh Phuoc.

#### Provider stock audit

For all providers who had anti-malarial drugs and/or malaria RDTs in stock on the day of the visit (25 providers), a short anti-malarial and malaria RDT stock audit (English version shown in Additional file 5. Provider antimalarial/RDT stock form) was also conducted either before or after the in-depth interview, according to provider preference.

### Data analysis

#### Key informant interviews

After all interviews were conducted, researchers (IC and HNTT) developed codes based on interview guides and perceptions gained from interviews. All audio recordings were reviewed and themes and representative quotes were identified and recorded in Atlas TI™ (Version 7.5.10, GmBH) (English by IC, Vietnamese by HNNT). All quotes in Vietnamese language were translated to the English language, and themes were identified and recorded in Atlas TI™ (by IC). All themes collected from key informant interviews were organized by code, and their frequency of appearance was documented.

#### Patient, provider and at-risk individual interviews

After all interviews were conducted, a researcher (HNTT) developed codes based on interview guides as well as perceptions gained from interviews, and discussion and review with researchers present during field interviews. Each interview was transcribed word-for-word, transcripts were uploaded to Atlas TI™, and organized by code. Thematic analysis was then conducted, and representative quotes were translated into the English language.

#### Provider stock audit

For the anti-malarial and malaria RDT stock audit, the anti-malarial active ingredients, RDT types, manufacturers, formulas and packaging details, retail prices from outlets to clients, and wholesale prices from distributors to outlets were collated in Microsoft Excel (2010 version, Microsoft).

## Results

A total of 61 interviews were conducted. The 11 key informant interviews were conducted across seven NGOs and government organizations in Hanoi, illustrating expert perceptions of risk factors for malaria nationwide, as well as the composition of the private sector in malaria endemic areas in Vietnam. All key informants approached (11/11) agreed to be interviewed. In Binh Phuoc and Kon Tum, 37 private outlets were approached, 30 of which agreed to participate in the study (outlet types detailed in Table 2). Non-participants refused because were not willing to talk about malaria, refused to answer questions on anti-malarials, were too busy or not available at the time of visit, or did not provide relevant information even though they were willing to participate. Most of the private pharmacies included in this study (10/13) were registered with the Department of Health and, therefore, permitted to provide anti-malarials to customers with a prescription. The same ten pharmacies also have a basic pharmaceutical license, received following completion of a 12–18 month training course. Most of the private clinics included in this study were registered (8) whereas some were not registered (2). Nine interviews with patients, and 11 interviews with at-risk individuals, all of whom were mobile workers, were also conducted (Table 2); all patients and at-risk individuals approached (20/20) agreed to be interviewed.

**Table 2:** Overview of interviews with providers, patients, and at-risk individuals

Individuals interviewed	Binh Phuoc Province	Kon Tum Province
Providers		
Pharmacy operators	6	7
Private clinic operators	5	5
Grocery shop operators	4	3
Total providers	15	15
Patients and at-risk individuals		
Malaria patients	4	5
At-risk individuals	6	5
Total individuals	10	10

### Key informant perspectives on the context of malaria elimination in Vietnam

Key informants described Vietnam’s commitment to malaria elimination by 2030 (Decision No 4717/QĐ-BYT, issued 2014). Recent plans focus on the implementation of test and treat strategies in both public and private health facilities (described in Decree No 1920/QĐ-TTg issued by Prime Minister in 2013), including requirements for rapid case reporting (Decision No 4717/QD-BYT, issued by MOH in 2014). Although these objectives are written in policy, at the time of interview, a key informant noted that specific plans to address private sector treatment, diagnosis and case reporting had not been developed:

“*On paper… they have some activity [plans] to try to educate the private sector into the malaria one, try to promote for public and private partnership, but they do not have any [activities] currently… That is the main malaria topic, they are trying to involve and engage private, but there are not yet plans*.”

—KI8

Consistent with these priorities described in national level policies, key informants expressed consensus that to achieve malaria elimination in Vietnam, the biggest priorities for the malaria program are to target treatment to vulnerable populations in remote locations, and to strengthen surveillance systems. Suggestions for targeting treatment to vulnerable populations included the potential to bring malaria services to people, for example through village health workers, mobile teams, or malaria posts placed either at the border or on a main road leading to the forest. Key informants also noted that surveillance and reporting could be improved by introducing electronic record systems to replace paper-based reports, which could help to provide the means for rapid response for detected cases, and also inform the supply chain for anti-malarials if needed.

### Key informant perspectives on malaria risk factors nationwide

Throughout Vietnam, key informants described the mountainous areas in the central highlands as having the greatest risk of ongoing malaria transmission. They described malaria as a disease of the rural poor in remote areas, often comprising of ethnic minorities. Migration, travel and forest working were also identified as risk factors for malaria. Migrants included those flying returning from work in Africa, travelling across international land borders between Cambodia and Laos, or migrating internally from north to south for seasonal work in the forest. Migrants introduce the risk of importing malaria to receptive areas, which, if not detected quickly, could potentially escalate into an outbreak. Forest workers were characterized as those working on plantations, construction, farms, mines (including gold mines), as well as fishermen. Within this group, the most vulnerable included small-scale farmers and forest goers, as well as individuals working for illegal mines, because other forest jobs (including construction and official mines) sometimes include the provision of healthcare on site. Forest workers present challenges for malaria elimination in being the most difficult to reach, particularly those working in illegal mines that are hidden and trying to escape detection from public authorities.

### Key informant overview of public and private providers in malaria endemic areas nationwide

Key informants provided an overview of both public and private facilities where people seek care for malaria, and that furthermore, self-treatment is a common practice in Vietnam:

“*Self treatment also very common in Vietnam, you know. So [in] everyone’s household, they have some basic types of medicine at their house already, so when they get fever, or minor illness, they treat themselves, that’s very common practice… People treat themselves for minor illness, and if it doesn’t work, they seek care*.”

—KI10

Public facilities included district and provincial hospitals, the commune health station (CHS), and village health workers. The CHS was described as the most common source of care for serious or severe diseases, and the main source of care for malaria in the public sector. At the CHS, malaria diagnosis is provided using microscopy, and treatment and diagnosis are free of charge. The CHS also provides standby drugs to people travelling to areas at risk of malaria, provided that these areas do not have documented drug resistance following Decision No 3232/QD-BYT, issued in 2013.

Key informants expressed that the private sector has a strong role in rural areas at risk of malaria. Private facilities for malaria care included private pharmacies, private clinics, grocery stores, and mobile vendors. Reasons for accessing the private sector rather than the public sector included convenience, improved reputation and quality of service, in particular a need for trust between providers and their clientele.

Pharmacies were described by key informants as the usual first point of care for mild illnesses in very rural areas. Generally, rural pharmacies are operated as a family business with multiple family members involved in operations. Pharmacies serve both local and migrant customers, given their ability to sell medication to all/any customers regardless of insurance coverage. Individuals working in pharmacies do not receive training for malaria diagnosis and treatment.

According to national policies, registered pharmacies are authorized to provide anti-malarials following prescription by medical doctors (Policy Decision #4718, Circular No 1517/BYT-KCB/2008, and Pharmaceutical Law 2016). Key informant interviews revealed that this was an area of confusion; only one key informant recognized that pharmacies are allowed to sell anti-malarial drugs, and several key informants had the misconception that pharmacies are not legally allowed to sell medicines for artemisinin-based combination therapy (ACT).

Private clinics are community level clinics often run by doctors working in the public sector after work hours, either early in the morning or in the evening. These individuals are the only link between public and private healthcare providers mentioned, as there are no formal public–private partnerships widely used in Vietnam. Private clinics usually offer basic curative care, including infusion, injection, and medication, and are legally allowed to provide treatment for non-complicated *P. falciparum* malaria infection, as well as malaria infection in pregnant women (Policy Decision No 3232/QD-BYT/2013). Key informants stated that only doctors in registered private clinics are authorized to diagnose and treat malaria cases, and that sometimes these providers offer anti-malarial drugs that they acquired from public facilities.

Grocery stores were described as highly informal outlets located in remote and border areas, usually operated by retailers without health training. They sell basic groceries, medicine and other household items, their clientele are a combination of local customers and migrants, particularly for shops near border areas. Officially, grocery outlets are not authorized to offer either malaria diagnosis or treatment, but can sell preventive items such as insecticide-treated nets, repellents, and coils.

Mobile vendors were described as highly informal, common sellers of basic medications, which are stocked by demand. Key informants mentioned that mobile vendors are well known by the community, and visit at fixed times and locations known by the community.

### Private provider perspectives and modes of operation in Binh Phuoc and Kon Tum

#### Stocking practices

Private pharmacies, clinics, and grocery store employees were interviewed in field sites. Three mobile vendors were identified, but were not interviewed because they reported that they did not stock anti-malarials. In outlets screened, anti-malarial medications were found in stock at 25 of the 30 outlets surveyed, most commonly consisting of quinine, chloroquine, and then ACT (Table 3). For context, the first-line treatment for *P. falciparum* malaria is dihydroartemisinin–piperaquine (3 days), first-line treatment for *P. vivax* malaria is chloroquine (3 days) and primaquine (0.25 mg/kg for 14 days), first-line treatment for mixed infection is dihydroartemisinin–piperaquine (3 days) and primaquine (0.25 mg/kg for 14 days), and second-line treatment for malaria is quinine (7 days) and doxycycline (7 days) or clindamycin (7 days).

**Table 3:** Antimalarial drugs and malaria rapid diagnostic tests from stock audit

Products	Manufacturer	Formula/packaging	Retail price (from outlets to clients)	Wholesale price (from distributor to outlets)	Outlet types
Antimalarial drugs					
Quinine sulphate (250 mg)	Mekophar Vietnam	Tablet 180T/pot	1200–1500 VND/tablet ($0.05–$0.07 USD)	180 VND/tablet ($0.01 USD)	5 clinics, 4 pharmacies and 2 grocery stores
Chloroquine phosphate (250 mg)	Mekophar Vietnam	Tablet 200T/pot	500–1500 VND/tablet ($0.02–$0.05 USD)	180 VND/tablet ($0.01 USD)	3 clinics, 3 pharmacies and 2 grocery stores
Arterakin (dihydroartemisinin 40 mg + piperaquine phosphate 320 mg)	Phabacor Vietnam	Tablet 10 T/1 blister pack	2000 VND/tablet ($0.09 USD)	200 VND/tablet ($0.01 USD)	3 clinics, 3 pharmacies and 1 grocery store
CV8 (dihydroartemisinin (32 mg) + piperaquine (90 mg) +trimethoprim (90 mg) +primaquine phosphate (5 mg)	OPC Vietnam	Tablet 8 T/1 blister pack	1200 VND/tablet ($0.05 USD)	No information given regarding source/price	2 private clinics and 1 pharmacy
Artesunat artesunate (50 mg) monotherapy	Phabacor Vietnam	Tablet 12T/1 blister pack	Offered freely to clients	Reportedly obtained from CHS	1 private clinic in Kon Tum
Malaria rapid diagnostic tests					
Malaria Ag P.f/P.v	Standard diagnostic	N/A	15,000–35,000 VND/test ($0.66–$1.64 USD)	3500–7000 VND/test ($0.15–$0.31 USD)	2 private clinics and 1 pharmacy

Conversion to USD: 22,727 VND/$1 USD (rate on August 30, 2017)

Expired anti-malarial drugs were found in 3/25 outlets, and in these cases, outlet operators emphasized that they had no plans to sell expired products but had simply not yet disposed of them. One of the expired drugs found was artemisinin monotherapy, potentially left over from before these were banned in Vietnam. RDTs were only found in stock in 3 of the 30 outlets audited, some of which were expired (June 2015). Only one type of RDT was found: Malaria Ag P.f/P.v by Standard Diagnostic.

The five outlets without stock on the day of the survey reported that they had recently sold out of quinine and/or chloroquine, the anti-malarials that are typically available at their outlets. Providers also mentioned that they tended to stock anti-malarial drugs more commonly during the rainy season (April to September), and that the time of study (December) was during low malaria season. Only one outlet reported sale of any anti-malarial within the past 7 days; Arterakin.

When providers were asked about where they obtain supplies, they were generally hesitant to disclose details but stated that they maintain stock levels using telephone orders from pharmaceutical distributors in provincial capitals or regional companies. Some providers reported receiving supplies from the CHS, either through family or friends who work there, or because they themselves work there.

Grocery store operators expressed that they often sell drugs based on their own personal experiences, or based on advice from pharmacists. They source medication from pharmacies in larger cities, often pharmacies in which they have a familial or otherwise personal connection with:

“*I sell drugs based on my personal experiences, I have no medical training. At the beginning I usually went to city to purchase goods for my grocery and my neighbour asked me help to buy some medicine for them. Since then I have stocked simple medicines*.”

—A grocery shop owner, Binh Phuoc

### Private provider perceptions of anti-malarial laws

Although registered pharmacies are authorized to provide anti-malarials following prescription by medical doctors (see ‘Provider Overview’ section), there may be a disconnect between national policies and local government understanding, and thereby enforcement of these policies. Pharmacy operators described recent inspections by the local Department of Health, where financial consequences and license suspensions were imposed for stocking anti-malarial medications:

“*Everything related to malaria has been banned from the private sector and therefore any malaria care at a private pharmacy or clinic is illegal*.”

—A pharmacy operator, Kon Tum

“*The DOH conducts an annual inspection of all pharmacies and clinics in this province. If the outlet is stocking any malaria medication they face fines and/or license suspension or loss*.”

—A pharmacy operator, Binh Phuoc

### Private provider diagnostic and treatment practices

Many of the private providers interviewed expressed that they diagnose malaria symptomatically, which may be consistent with stock audit findings on the low availability of RDTs in outlets audited. For pharmacies, only one outlet audited had RDTs in stock. Providers from private clinics also described that they did not trust RDT results:

“*I only conduct diagnosis based on signs and symptoms reported by my patients. I do not test. But previously there were some patients who visited me, I tested with a malaria rapid test and got negative results. No drugs were effective until I used Chloroquine, then the fever disappeared*.”

—A private clinic employee, Kon Tum

For treatment practices, a pharmacy operator in Kon Tum mentioned that they dispense anti-malarials with a variety of other medications:

“*If a customer come with symptoms of fever and cold for 3 continuous days I give this package consisting of flu drugs, fever reliever, and chloroquine. But normally I start with a basic drug such as paracetamol. If the fever continues, then I give them [artesunate]*.”

—A pharmacy operator, Kon Tum

### Malaria training among private providers

Many of the private providers interviewed reported having received little to no official training on malaria, or if they did receive training through medical school, had not received updates on malaria treatment and testing since then. Some providers participated in trainings, however these were integrated programmes that were not specific to malaria, and providers felt they did not offer sufficient information on malaria. As a result of the lack of formal training on malaria, private providers rely on instinct, experience and self-taught methods to provide malaria care to their clients/customers:

“*I also work at a CHS, so I’m invited to the district health centre for annual malaria training, but I haven’t had time to attend, so I only received the documents and read them myself to treat patients*.”

—A private clinic employee and CHS worker, Kon Tum

“*I learned about malaria treatment by myself, but do not offer any official treatment regime. I have cured patients based on my experience*.”

—A pharmacy operator, Binh Phuoc

### Private provider motivations

When asked about their motivation as providers, respondents expressed that profit remains their primary incentive. In the context of Vietnam’s low and decreasing malaria burden, providers explained that they were less motivated to stock malaria products, given a relatively low and seasonal demand:

“*As malaria is not a big health issue, we do not stock anti*-*malarials regularly. However, if there is a malaria patient asking for drug, I can provide it to them*.”

—A pharmacy operator, Kon Tum

Providers also expressed that most of their clients are regular clients, and that they are motivated by altruism, given their role as caregivers within the community:

“*I am happy when I saw my patient recover or happy when someone come back to me and say thank as I treated his/her well*.”

—A pharmacy operator, Kon Tum

### Links between the public and private sector

There were no formal links between the public and private sector in Vietnam, confirming that malaria cases treated through the informal private sector are not reported to the public surveillance system. However, there are informal links between the public and private sector, a significant one being that many private clinic providers work at the CHS during the day. Both pharmacy providers and those in private clinics also described referral practices to the public sector, which they explained were for serious illness, as well as in cases where the treatment they offer does not resolve symptoms:

“*We generally will make referrals to the public facilities if the patient is seriously ill, or if our offered treatment does not work well*.”

—A pharmacy operator, Kon Tum

### Communication channels for private providers

The providers interviewed were asked about popular methods of communication, as providers may be willing to use these methods for reporting malaria cases to public facilities. The providers interviewed stated that the phone was the most popular and main communication method. Almost all interviewed private providers also had internet access, but mentioned that access was not stable:

“*I am not often searching information on the internet, also we only have 3G internet which is not stable.*”

—A private clinic employee, Binh Phuoc

Private clinic operators communicate with each other and nearby pharmacies, using text messages and “Zalo” to share information about government inspections, refer clients for drugs they do not have in stock, and more.

### At-risk individuals in field sites: care-seeking behaviour

In the field sites selected for study, patients described private pharmacies, clinics, and grocery stores as their main first point of care, a finding consistent with those found in Key Informant interviews. Patients described these outlets as being relatively accessible, usually being located within 1–5 km from their community. The CHS, on the other hand, was approximately 3–12 km from the communities visited, and 30 km from the forest where interviewees worked. Patients mentioned that they did not use the CHS due to ‘hidden’ costs such as transportation and consultation fees, and furthermore, treatment was only free for those with insurance:

“*Just 4* *months ago was my fourth time having malaria. I did not test, but I knew [I had malaria] because the symptoms were similar. I went to the commune health station once. They gave me CV8 [DHA*-*PIP] for treatment. I had to pay for consultation and services. But I did not recover so I went to the newly opened private clinic close to my home. They charged me the same amount for the same drugs I got from the CHS before [showed the drugs]. So I think that nothing is free.*”

—A malaria patient, Kon Tum

Patients and forest goers described that they preferred to visit pharmacies and/or private clinics due to convenience, patient-appropriate hours of operation, and perceived higher quality of care including provider friendliness.

“*If you get ill on Sunday or Saturday, you have to wait until Monday to get treatment from CHS, if you wait until then, you will die. I normally go to the pharmacy first and ask for treatment. If the symptoms do not go away, I go to CHS later*.”

—A malaria patient, Kon Tum

“*I have never visited the CHS. If I am ill, I go to the private clinics close to the road and ask for treatment. I’m scare to go to the CHS. For any disease they will take my blood for testing* –*they recommend that I do not know*.”

—A malaria patient, Kon Tum

Patients also described self-treatment as a common practice, another finding consistent with perceptions noted by Key Informants. In some instances, individuals in the community gave anti-malarial medications they had on hand to their friends, or went to the pharmacy to purchase anti-malarials for their friends:

“*My friend told me what malaria symptoms are and gave me some medicine that he took before, and told me to visit Dr… a private provider, for treatment. Fortunately, after taking his medicine my symptoms went away*.”

—A local forest goer, Binh Phuoc

In other cases, mobile workers reported travelling with medication on-hand, given difficulty accessing health care on or near farm or forest sites. Medications chosen were usually cocktails of drugs to treat common illnesses, including malaria, to be used while they are working in the field for days or weeks at a time. These cocktails were often purchased from rural grocery shops close to home or near entry points into the forest:

“*In the forest, we have no time to leave for testing and treatment, so we normally bring some drugs with us for when we get malaria in the forest. In Cambodia, it is easier to purchase these medicines*.”

—A migrant forest goer, Kon Tum

Patients and forest goers mentioned that payments were not an impediment to accessing treatment from private providers. While cash was the most popular way of payment for treatment, some providers were willing to accept trade payments and/or credit or deferred payments. However, credit/deferred payments were only an option for patients who were regular clients to the provider:

“*When we do not have money, we can still come to get drug and pay later. We know each other very well. The doctor knows everyone in this village*”

—A local forest goer, Kon Tum

“*We have only cash and pay in cash, we travel frequently from this area to others, the owners do not know who I am, how they can give us treatment without money*”

—A migrant forest goer, Kon Tum

### At-risk populations in field sites: characteristics, perceptions of malaria, and communication channels

The at-risk individuals interviewed included forest goers (three local, two migrant), three plantation workers, and three farmers who spent at least one night within the last 3 months within or near a forest in the study area.

Forest goers comprise of men, ages 20–50 years, who travel to the forest without their families. There are two general types of forest goers; those that are local, and those that are migrants. Local forest goers are typically ethnic minorities (X’tieng, Gia Lai, Ede, Bana, Van Kieu, Tay and Nung) that live in villages close to forests. They typically stay in the forest for a few days during the dry season, collecting supplies and sometimes crossing the border. They visit the forest primarily during the dry season, and reported earning between 1.2 and 2.8 million VND ($60 to $110 USD) per month from the sale of items forest items. Migrant forest goers tend to be Kinh, the majority ethnic group in Vietnam, and travel from other provinces and/or across international borders. They reported staying in the forest for up to a month at a time, returning to their home province a few times a year. Migrant forest goers reported earning between 3.5 and 5.7 million VND ($150 to $270 USD) per month.

Ethnic minorities face a number of challenges, including living in remote, rural areas with limited access to care, and limited access to information. Most of them are poor, living in bamboo houses and following traditional lifestyle habits, for example, not using mosquito bednets. They also face challenges with language barriers with the Vietnamese majority group. Money and family are primary motivators for forest goers:

“*If we are lucky, for example, hunting many animals, we can earn 7*–*8 million Vietnam Dong* [$308–352 USD] each *time for a group of 5*–*6 people*.”

—A local forest goer, Kon Tum

Forest goers reported bringing medicines to the forest, to treat common illnesses. Many forest goers in border districts also reported easy access to malaria drugs through outlets across the border, in Cambodia. Both local and migrant forest goers reported avoiding forest rangers, due to the risk that encounters will require bribe payments from the rangers.

“*We bring some drugs to treat common illnesses, including malaria. We purchase these drugs from a private clinic or pharmacy or, more frequently, from a non*-*health outlet* — *like an FMCG [Fast Moving Consumer Goods; mobile vendor].*”

—A migrant forest goer, Binh Phuoc

The other main group at risk for malaria is small-scale farmers, who work on small, informal farms or plantations in or near the forest. They usually travel to town on a weekly basis to restock supplies at home and local markets.

#### Perceptions of malaria

Individuals at risk of malaria mostly perceived the risk of malaria to be low, and understood that malaria is transmitted by mosquitoes. However, myths and misconceptions exist; that malaria only infects unhealthy individuals, or that malaria is transmitted through wind or water:

“*No, I am not at risk as malaria is not a problem and can be treated easily, also it only infects those who are not healthy*.”

—A farmer, Binh Phuoc

At-risk individuals expressed a very basic knowledge of malaria symptoms, described to be similar to fever or cold. There was little knowledge or use of RDTs, and very limited knowledge of malaria drugs; patients explained that they received very little information from providers related to diagnosis and/or the medication provided.

Treatment adherence rates were also reportedly low; most interviewees who had been diagnosed with malaria previously reported that they never completed the full course of drugs acquired from their health provider. Both providers and at-risk individuals described patients taking the medicine until their symptoms went away (even when diagnosed at the CHS), often buying partial courses of treatment due to financial constraints:

“*They only purchase medicine for 1 or 2 doses, then if the symptoms persist they come back and order more. There are many reasons, such as no money, but it is mainly due to their knowledge*—*they do not think it is necessary*.”

—A private clinic employee, Kon Tum

At-risk individuals described two main malaria prevention methods: hammocks and mosquito nets. Preventive measures are not used consistently; a farmer explained that not enough nets are available on the farm, and a forest goer expressed a misconception that drinking alcohol can prevent mosquito bites:

“*We normally sleep after drinking so we often do not use the net, mosquitoes do not bite drunk people*.”

—A local forest goer, Kon Tum

Hammocks nets were less common; some at-risk individuals had never heard of them, others stated they were hard to find:

“*I bought this hammock net in Cambodia. I cannot find this here in Vietnam. Even in Cambodia, you have to know where to buy this, it is not common, it is mostly used for the military*.”

—A migrant forest goer, Kon Tum

#### Communication channels accessible to high risk groups

Given the remote, rural areas where they live and work, forest goers and small-scale farmers reported having relatively limited access to communication channels. Health information was usually provided to high-risk populations by village health workers through traditional communication channels managed by local authorities, such as the commune loudspeaker announcements and community hall signboards, in addition to word of mouth from friends, family and local leaders.

Mobile phones were noted as the main source of communication, however coverage was very limited in forests and remote farm areas. Additionally, border crossing posed further barrier to mobile phone use, as migrant forest goers expressed the need to buy new SIM cards when crossing borders to Cambodia or Laos.

The internet was seldom used to access information, and only used by a few forest goers and small-scale farmers interviewed. Television was used for information, but most at-risk populations do not own televisions, and viewed television at cafes or at their neighbor’s place.

## Discussion

Malaria elimination programmes share a common goal of providing testing, treating, and tracking for all infections. Currently, all three of these goals are threatened by practices in the private sector, the main first point of care in rural, malaria endemic areas [3, 4]. This study is the first to describe and characterize the private health sector accessed by high-risk populations of malaria in Vietnam, as well as their clientele’s characteristics and care-seeking behaviour, providing crucial insights for the Vietnamese malaria programme as the country prepares for malaria elimination.

The results from this study suggest that improved malaria treatment and diagnostic practices should be targeted to private pharmacies, private clinics, and grocery stores in remote, malaria endemic areas. Currently, treatment is given on the basis of symptoms, leaving no room to discriminate between *P. falciparum* malaria, *P. vivax* malaria, and other non-febrile illnesses. Ideally, this practice should be replaced with an effective training programme on RDT use, accompanied by subsidized provision of RDTs, as providers otherwise do not receive training on malaria treatment and diagnostic practices. There is also a need to incorporate a surveillance and referral mechanism within the informal private sector; current cases remain unreported, preventing the malaria programme from tracking, and thereby responding to cases and outbreaks. The findings in the study suggest that pharmacies and grocery stores do not have formal links with the public sector, and furthermore, that they prefer to communicate using mobile phones. Thus, mobile phones offer a potential method for case reporting and referral to public facilities. Also, the study found that doctors at private clinics commonly work at the CHS in the public sector during the day, such that these doctors may be asked to report cases to the CHS during their day job.

An interesting gap found in this study is the misconception of national policies, that registered pharmacies are allowed to provide anti-malarial drugs provided they are accompanied by a formal prescription. This gap was noted through contradictions in key informant interviews, as well as provider interviews in the field, who noted that they would be fined if they carry anti-malarial drugs. Efforts should be made to clarify this national policy at the provincial level.

Among malaria patients and at-risk individuals, this study identified a few key factors may threaten efforts to eliminate malaria. First, individuals cited a limited understanding of malaria transmission and prevention, including misconceptions that malaria is spread by wind or water, and that mosquitoes do not bite individuals who are drunk. Second, individuals reported not completing their courses of anti-malarial medications, a dangerous practice that threatens the further emergence of drug resistance [22]. Third, individuals reported that they often stock medications at home and practice self-medication, a practice that has been documented previously, and was also noted by KIs [23]. Education is needed among at-risk populations to improve on their knowledge and awareness of malaria, including encouraging the use of preventive measures, communicating the importance of acquiring diagnosis using a test, and emphasizing the importance of completing full courses of treatment.

At-risk individuals also expressed that insecticide-treated hammock nets were difficult to acquire, a preventive measure that may be important in Southeast Asian settings, where many Anopheline species are outdoor biters. Further research is needed, both to assess the efficacy of insecticide-treated hammock nets, and to explore additional methods to combat outdoor-biting mosquitoes, particularly for individuals who sleep outside.

One area that must be emphasized is that reaching these populations will require an engagement strategy to optimize delivery, as in the absence of effective delivery, even the strongest interventions may be lost to poor adherence and uptake. This study provides insight for engagement strategies, finding that providers care about both profit and altruism, and that at-risk individuals mostly comprise of men staying in the forest for days or weeks at a time, often drinking with their peers in the evening. These insights can be used in malaria elimination programme plans, as a foundation for how to reach and motivate providers and at-risk individuals, promoting early diagnosis, treatment, and adherence. Indeed, these types of insights have laid the foundation for behaviour change communication (BCC) strategies in many countries including Cambodia and Myanmar, where ACT and RDT use have been piloted using BCC strategies, successfully creating product awareness and encouraging uptake [24, 25]. In these studies, the authors emphasize the importance of BCC for RDT use, which requires complex messaging when targeted to providers in the informal private sector. In Vietnam, the use of BCC approaches have not been documented for malaria control and elimination, but have shown to be effective for injection drug users at risk of HIV [26]. Malaria elimination efforts in Vietnam can likely be accelerated through BCC, targeting improved treatment, diagnosis, and reporting practices to private providers and at-risk individuals.

This study is limited to inference drawn from qualitative insights across a small group of respondents in target areas, and is not nationally representative. However, given the concordance between providers noted by key informants and results found in the field, the findings on private providers and at-risk populations in this study are likely to be largely generalizable across malaria endemic areas of Vietnam. However, the care-seeking behaviour found in this study may be more specific to the locations studied, given different compositions of ethnic minorities and their respective traditions, as well as different migration patterns, lending to subtle differences between at-risk populations in the two provinces studied. This study did not include at-risk populations in large plantations and worksites that provide their own healthcare, nor did it address the issue of international migrants from Africa, which are gaps that should be addressed by future studies.

## Additional files

**Additional file 1.** Semistructured Interview guide: key informant.

**Additional file 2.** Semistructured Interview Guide: private provider.

**Additional file 3.** Semistructured Interview Guide: malaria patient.

**Additional file 4.** Semistructured Interview Guide: individual at risk of malaria.

**Additional file 5.** Provider antimalarial/malaria rapid diagnostic test stock form.
