# Supplementary material for: Malaria risk factors and care-seeking behaviour within the private sector among high-risk populations in Vietnam: a qualitative study
Source: Malar J. 2017 Oct 16;16:414. doi: 10.1186/s12936-017-2060-0 (PMC5644094; doi:10.1186/s12936-017-2060-0)
Supplement: Supplementary file 1 — Additional file 1. Semistructured Interview guide: key informant. [file 12936_2017_2060_MOESM1_ESM.docx]

**Additional file 1. Semistructured Interview guide: key informant**

Duration of interview: ______ minutes Interview code number: ______

Name of organization: _________________________________________________________________

1. I would like to learn about you and your organization.
   - How long have you been working here?
   - In your work, have you been involved in malaria control in Vietnam? (If yes, could you tell me a bit more about these activities? Where did they take place?)
   - Have you worked on healthcare outside of public health facilities before, for any disease? How about for malaria specifically?
2. Please tell me about malaria in Vietnam.
   - Who is at risk for malaria in Vietnam? (Locations, populations)
   - Could you tell me about where people seek care for malaria in Vietnam?
3. What are the current major sources of funding for malaria case management, including government and non-government funding sources? Please describe.
4. The most recent malaria case management guidelines for Vietnam were published in 2013. Have any new malaria case management policies been implemented? This includes any changes to national treatment guidelines for malaria.
   - What are the current strategies to increase access to testing?
   - What are the current strategies to increase access to treatment?
   - What are the current strategies to improve knowledge and practices or providers in the public sector?
   - Have there been any policy changes related to the *cost* of testing and treatment in the public and/or private sectors? Please describe.
   - Have there been any important changes in malaria pharmaceutical regulation? This includes changes to the regulation of malaria medicines and diagnostics, new regulations implemented, efforts to reinforce existing regulations and bans on medicines. Please describe.
   - Have there been any policy changes related to who is qualified to test and treat suspected malaria, including community health workers, or segments of the private sector? Please describe.
5. What are the main challenges currently facing Vietnam to ensure that suspected cases of malaria receive a diagnostic test and appropriate treatment?
   - Any challenges related to ensuring that fever cases receive a malaria blood test?
   - Any challenges related to ensuring that positive cases receive the national first line treatment?
   - Any challenges to ensuring continuous stock of malaria case management commodities at the places where people seek care including recent or persistent disruptions to supply chains?
   - What do you see as the main priorities that must be addressed to improve malaria case management in Vietnam?
6. What are the main challenges for ensuring that suspected cases of malaria receive a diagnostic test and appropriate treatment, specifically among mobile and migrant populations residing in the Central Highlands region?
   - Where do these populations seek and receive fever treatment?
   - What is needed to better serve the fever case management needs of these populations?
7. Do people in Vietnam look for malaria care outside of public health facilities?
   - If yes, what kinds of facilities are these? Who has malaria treatment available? Whether or not they are legally allowed to) (Probe: drug shops, itinerant drug vendors, general retailers, not-for-profit facilities, private companies giving care to employees). Could you please tell me a bit about what usually happens when people look for care for malaria from one of these providers? (Probe: antimalarials, referral, rapid diagnostic test?)
   - If no: how about other healthcare outside of public health facilities? What kinds of facilities are these? What kinds of diseases do they treat?)
8. Private providers in Vietnam
   - I would like to call these people providing healthcare outside of the public sector, ‘private providers’ for the rest of our interview.
   - Could you please tell me about how you think private providers operate
     1. Where do they get their medicine from? How does the supply chain work in Vietnam?
     2. Are they at all regulated, for example, do they ever seek registration with any organizations or authorities? Is there official certification or training that they seek?
     3. Do private providers liaise with public facilities at all? Are there any mechanisms offered by public facilities, for example training, supplies, or otherwise that give private providers a reason to work with public facilities?
     4. How do you think private providers decide what to stock?
     5. What kind of clients do you think go to these private providers for healthcare? Are there specific locations, people with certain types of jobs or socioeconomic status, that seek healthcare from private providers?
   - Do you think the quality of care offered by private providers could be improved? If so, what are the main problems you can think of?
   - Could you please tell me about what you think private providers care about, to consider their business a success? (Probe: profit, reputation, being helpful / important for the community)
     1. Are there specific areas in Vietnam where you think that the private provider plays a strong role in healthcare? (Probe: are there any districts you can think of?)
9. Please describe any public-private partnerships or initiatives to engage the private sector in malaria case management.
   - Are there any new initiatives to improve private provider knowledge and practices? Please describe.
   - Are there any initiatives to strengthen regulation and oversight of the private sector? Please describe.
10. Please describe any initiatives to strengthen national supply chains that would have effect on continuous supply of malaria case management medicines and rapid diagnostic tests specifically.
