# Supplementary material for: Malaria risk factors and care-seeking behaviour within the private sector among high-risk populations in Vietnam: a qualitative study
Source: Malar J. 2017 Oct 16;16:414. doi: 10.1186/s12936-017-2060-0 (PMC5644094; doi:10.1186/s12936-017-2060-0)
Supplement: Supplementary file 2 — Additional file 2. Semistructured Interview Guide: private provider. [file 12936_2017_2060_MOESM2_ESM.docx]

**Additional file 2. Semistructured Interview guide: private provider**

| **Census Information. *Interviewer completes this section for all outlets.*** | | | |  |
| --- | --- | --- | --- | --- |
| **Outlet ID** [___\|___\|___] | | | |  |
| 1. Today’s date (dd/mm/yyyy) | [___\|___]-[___\|___]-[_2_\|_0_\|_1_\|_5_] | | |  |
| 2. Interviewer’s name  [________________________________________________] | 2a. Interviewer’s code  [___\|___] | | |  |
| 3. District  [__________________________________________________________________] | | | |  |
| 4. Village/Street  [__________________________________________________________________] | | | |  |
| 5. Type of Outlet  Drug store  Private provider  General retail | | Traditional healer  Itinerant medicine seller  Other ***(specify)***  [_______________________] |  | |

+ Drug store: non-licensed and non-registered, could be at individual home or at market.

+ Private provider: Who working for public health facilities, but practicing as private provider at home, outside of working time and non-licensed or non-registered

+ General retail: Retail outlets sell fast moving consumer goods and may stock a small range of medicines, mostly for normal diseases.

+ Traditional healer: Who provides counseling and examination at home

+ Itinerant drug vendor: Who sell medicines included traditional medicine, moving around districts from this commune to another commune in period (twice per week), located at community market.

1. I would like to learn about your facility.

- How long have you been working here?
- Do you live around here? How long have you been living here?
- Who comes here for services?
  - How about health services, what kind of problems do people look for help with here?
  - Which kinds of people come here for health services? (Probe: gender, age, socioeconomic status)
- Where do you get your supplies and commodities from?
- How do you decide what to sell in your facility? (Probe: Profit, what the client wants, what the government says, something else?)
  - Please tell me more.
  - Where do you get information from, on different products you can sell here?
- Have you worked with the public sector before, in government facilities, in the past?
- What about now, do you ever work with the public sector, with government facilities?
- Do patients ever come to you with a prescription from the public sector?
- Do you ever refer your patients to the public sector, to government facilities? If yes, when do you usually make the referral, under what kind of situation?

1. I would like to learn about malaria

- Is malaria a problem in this area?
  - If yes: who does it affect? Where do they go for treatment? Do they ever come here? What do you have for them? (Probe: how do you decide it is malaria? What do you give to the person?)
  - If no: has it always been like this? Is malaria a problem in other places in Vietnam?
- Have you ever gotten training on malaria case management? (If so, could you tell me more about this please? Who conducted the training? What were the main things they taught you?)

1. I would like to ask you some more questions about you and your facility

- Do you have a phone here? How about SMS messaging? Do you use it often? How much credit do you need to send a message or make a call?
- How about internet? Do you have access to the internet here in the shop? Do you use it much?
- Please tell me more about your work here. What do you like about working here?
- Is there anything more you could be doing to help your business, or your experience working here?
- Are there other facilities around here, that are similar to yours? Where are they, and how are they different from this one?
