# Supplementary material for: Malaria risk factors and care-seeking behaviour within the private sector among high-risk populations in Vietnam: a qualitative study
Source: Malar J. 2017 Oct 16;16:414. doi: 10.1186/s12936-017-2060-0 (PMC5644094; doi:10.1186/s12936-017-2060-0)
Supplement: Supplementary file 3 — Additional file 3. Semistructured Interview Guide: malaria patient. [file 12936_2017_2060_MOESM3_ESM.docx]

**Additional file 3. Semistructured Interview guide: malaria patient**

I work for a health NGO and we would like to ask you some questions about you and your malaria care It will take approximately 60 minutes – and so that I can remember everything we’d like to tape record our conversation. All your questions will be kept completely anonymous. We want to find out from you how we can provide better health services for people like you who work in the forest or on a plantation. Are you happy to start?

1. I would like to ask you about your recent malaria experience.

- When was the most recent time you were diagnosed with malaria? Was this your first time being diagnosed malaria?
- How many times have you been diagnosed with malaria to date?
- Is there anyone else in your family who has been diagnosed with malaria?

1. I would like to learn about the private provider who you sought malaria care from recently.

Which private provider did you seek malaria care from recently? Where are they? How have you learned about them? Was it far from your house? How have you accessed from your house?

- Can you please tell me about the reason you came to see the provider? (Probe: fever, convulsions, difficulty breathing, vomiting, diarrhea, excessive sleeping, anything else?)
  - How many days did this illness begin before you sought care from this facility/site?
  - Have you come to this facility before, for the same illness? (If yes, how long was that?)
- Can you please tell me why you chose to come here, instead of another place, to look for healthcare recently? (Probe: short wait time, closest facility to home, availability of different types of medicine, availability of services, the shop is open often, the provider is knowledgeable, the provider is nice to you, the facility is clean, services are inexpensive, I get to choose what to buy) – if several reasons, probe for the most important reason
- Is this the first place you went, to look for care for this illness?
  - If not, can you please tell me where you went for care before? Why did you decide to come here afterwards?
- Who else comes here for health services? Can you tell me which kinds of people come here? (Probe: gender, age, socioeconomic status)
  - What kind of problems do people look for help with here? (Probe: fever, convulsions, difficulty breathing, vomiting, diarrhea, excessive sleeping, anything else?)

1. I would like to learn about your experience with the provider

- When you came into the shop, about how much time did you have to wait before seeing the provider?
- How much time did the provider spend consulting with you?
- Can you please describe to me what happened during your consultation with the provider? How did they decide what kind of illness you or the person you are caring for has? (Probe: did they have a discussion with you, or examination of the body, or do a laboratory test?)
  - Did the provider tell you the name of the illness you have?
  - Did the provider explain to you what is wrong, and why you feel ill?
- Did you buy medicine from the provider?
  - If yes, can I see the medicine please?
  - How did you decide what kind of medicine to buy from the provider? (Probe: do you follow the provider’s recommendations, do you tell the provider what you want because already know what is wrong and what type of medicine you need, do you ask for certain medications because you heard they were good? If so, where did you hear about the medications?)
  - For the medication you got\do you feel comfortable and confident that you know how much medicine to take each day and for how many days to take it? (Probe: who, where, why, were you given any paper with information, do you intend to follow the instructions? Why or why not)
  - How do you decide how much medicine to buy from the provider? (Probe: does the provider decide, or does it depend on how much the medicine costs and how much you can afford, or something else?)
  - Can you tell me how much you paid for the medicine you bought?
- Did the provider tell you about any signs or symptoms you may see where you must come back? (If yes, can you please tell me what those are?)
- Did the provider tell you anything about coming back to the health facility for follow-up for non-emergency reasons? If yes, why would you come back?
- Has this provider ever told you to go somewhere else for care? If yes, can you please tell me more? Where did the provider tell you to go, and what kind of illness did you have? (Probe: fever, convulsions, difficulty breathing, vomiting, diarrhea, excessive sleeping, anything else?)
- What will you do if you do not get better, or get worse? (Probe: return to facility, go to other facility, go to traditional healer, go to other health worker or pharmacy, nothing just wait, don’t know)
- Are you satisfied with the care you got from the provider? (If no, is there anything you wanted more or, or less of, that would make you more satisfied with the visit? If yes, what was the most important thing to you for this visit?)

1. I would like to ask you a few questions about your community

- What kind of work do most people who live around here do?
  - Do they work or sleep outside?
  - Do you work or sleep outside?
- Do people around here travel to other areas to find work? (If yes, probe: what kind of work is that, how often do they travel, and when?)
- Do you live around here? How long have you lived there?

1. My last questions for you are about malaria. I would like to ask you a few more questions.

- Is malaria a problem in this area? If yes, who does it affect? Where to they go for treatment, do they come here or go somewhere else?
- For someone like yourself in the community where you live, how many times do you think a person will get malaria in the next 12 months?
- Can you describe the different types of people who seem to get sick with malaria most often in the community? For example, are their certain groups of people who have been infected with malaria that work together, live together or travel together?
  - Do these groups differ by gender, age, education or ethnicity?
  - Have you noticed that those who are higher risk for malaria interact with each other? If so, how?
- Where would you say the majority of individuals infected with malaria come from? (Probe: are they from the same village? Other countries?)
