# Supplementary material for: Malaria risk factors and care-seeking behaviour within the private sector among high-risk populations in Vietnam: a qualitative study
Source: Malar J. 2017 Oct 16;16:414. doi: 10.1186/s12936-017-2060-0 (PMC5644094; doi:10.1186/s12936-017-2060-0)
Supplement: Supplementary file 4 — Additional file 4. Semistructured Interview Guide: individual at risk of malaria. [file 12936_2017_2060_MOESM4_ESM.docx]

**Additional file 4. Semistructured Interview guide: individual at risk of malaria**

I work for a health NGO and we would like to ask you some questions about you and your work in the forests please. It will take approximately 60 minutes – and so that I can remember everything we’d like to tape record our conversation. All your questions will be kept completely anonymous. We want to find out from you how we can provide better health services for people like you who work in the forest. Are you happy to start?

Notes: This questionnaire will be used to interview individuals at high risk of malaria due to their work (and stay/sleeping) in farm/plantation settings. Interview question language will be slightly modified for these high-risk individuals as appropriate.

1. I would like to learn about you

- How old are you?
- Are you married? Do you have any children?
- Where do you normally live (commune/district/province)?

2) I want to talk to you about forest work

- How long have you worked in the forest?
- What type of work do you do in the forest? (Does it involved collecting plants, or animals or wood – if wood what type)?
- How did you find this work (through someone or…)?
- Do you work for someone else, or for yourself?
- How long (number of days) do you normally stay in the forest?
- Do you go to the same place each time?
- Is there a season where you go more often (peak months)?
- Do you or someone else take the products out of forest?
- Do you go to the forest alone or with friends?
- How do you spend your free time when you are IN the forest?
- What do you like most about the forest work?
- What do you not like most about the forest work?
- Is forest work dangerous?

3) I want to ask you about sleeping

- Do you sleep in the forest?
- Where do you sleep?
- Do you use a net?
- What type of net?
- What do you think about hammock nets? Do you like them, not like them and why?
- Do you get bitten by mosquitoes?
- What do you do about that?

4) I want to ask you about traveling

- How do you travel from home to the forest (bus, motorbike, truck…)?
- Have you worked in a neighboring country in the last year? If yes, provide details: when/why/how long?

5) I want to ask you about food

- What do you eat when you are in the forest?
- Do you cook or does someone else cook for you in the forest?
- Describe your typical largest meal of the day?

6) I want to know what you take with you to the forest

- What things do you take with you when you go into the forest? [List them]
- What is the most useful thing you take?
- What things do you buy inside or nearby the forest? From where?
- Do you usually visit the same place [shop]?
- Would you like them to stock anything which they don’t have right now?

7) I want to ask you about communications

- How do you communicate with your family when you in the forest?
- What is your typical cell phone credit balance when you go into the forest?
- If you work in Lao or Cambodia, how do you communicate with your family during these trips?
- How about internet? Do you have access to the internet? Do you use it much?
- What is your favorite radio station or TV channel/program?
- For radio do you listen to FM or AM?
- What is your favorite sport - why?

8) I want to learn even more about you!

- Who is your role model?
- What do you worry about?
- If you won a lottery pay-out of 5 million VND, how would you use it?
- Which type of alcohol do you drink usually?
- Which type of cigarettes or tobacco do you typically use?
- Any other drugs?
- Where/how do you spend your free time OUTSIDE of the forest?
- Who has the most influence in your life?
- Where do you see yourself in 2 years?
- How would you describe yourself to someone who does not know you?
- When you get paid, what is the first thing you spend your money on?

9) Can we now talk a little about malaria …

- Do you know how you get malaria?
- Do you feel at risk of malaria? (Why and why not)?
- Have you, or anyone you know, suffered from malaria?
- What are the symptoms of malaria?
- Do you know how malaria is prevented?
- If you have suffered from malaria, when was this?
- Please tell me about a recent time when you got a fever and sought care?
- Where did you go?
- Did you get test?
- What did you take for treatment?
- Do you carry anything into the forest with you to protect yourself from mosquitoes?
- Do you take any medicine into the forest with you to treat yourself if you feel feverish in the forest?
- What do you take?
- Where do you get these items?

10) How can we help?

Our organization provides health in Vietnam and we want to provide health services for you and people who work in the forest - what do you think would work best? Where would be easy for you to get to? What would you like us to provide?

Close: some discussion needed to inform access analysis. For example: ‘can you describe any restrictions which could affect a health organization from reaching people like you in the forest’?
