# Supplementary material for: Malaria risk factors and care-seeking behaviour within the private sector among high-risk populations in Vietnam: a qualitative study
Source: Malar J. 2017 Oct 16;16:414. doi: 10.1186/s12936-017-2060-0 (PMC5644094; doi:10.1186/s12936-017-2060-0)
Supplement: Supplementary file 5 — Additional file 5. Provider antimalarial/malaria rapid diagnostic test stock form. [file 12936_2017_2060_MOESM5_ESM.docx]

**Additional file 5. Provider antimalarial / RDT stock form**

| **Section 1: Screening & Eligibility** |  |
| --- | --- |
| S1. Do you have any antimalarial medicines in stock today?  1 = Yes ***Go to Section 2***  0 = No | [___] |
| S2. Are there any antimalarial medicines that are out of stock today, but that you stocked in the **past 3 months?**  1 = Yes ***Go to Section 2, question A.16***  0 = No  8 = Don’t know ***Go to Section 2, question A.16*** | [___] |
| S3. Are you offering any malaria diagnostic services or selling any malaria diagnostic tests here today?  1 = Yes **Go *to Section 3: Diagnostic Audit***  0 = No | [___] |
| S4. Are there any malaria diagnostic services or tests that are out of stock today, but that you stocked in the **past 3 months**?  1 = Yes  **Go *to Section 3: Diagnostic Audit, question D.9***  0 = No | [___] |

| **Section 2: Antimalarial Audit** |
| --- |
| ***A0. Read to the provider:***  Can you please show us the full range of antimalarials that you currently have in stock?  ***If the outlet has no antimalarials in stock cross-check screening results, then proceed to question A16.*** |

***Proceed to the antimalarial audit. Different antimalarial audit sheets will be used to record the antimalarial information based on the dosage form of the medicine.***

***Separate the antimalarials into two piles:***

- ***The first pile should contain all the antimalarials in the form of tablets, suppositories, or granules.
  Use the Tablets, Suppositories & Granules Drug Audit Sheet to record these.***
- ***The second pile should contain all the antimalarials in any form other than tablets, suppositories or granules. Use the Non-Tablet Drug Audit Sheet to record these.***

***If additional audit sheets are used, add these sheets after the ones provided and staple the questionnaire again.***

***All pages should be in order before you move onto the next outlet.***

***Number each audit sheet used in the spaces provided at the bottom of the page.***

|  | **1. Generic name** | | | | | **2. Strength**  **[__\|__\|__].**[__]mg  **[__\|__\|__].**[__]mg  **[__\|__\|__].**[__]mg | | **2a. Is this base strength?**  [__]  1 = Yes  [__] 0 = No  8 = Don’t know  [__]  ***If no, specify salt:***  [________________________] | | | **3. Dosage form**  1 = Tablet  2 = Suppository  3 = Granule  [___] | **4. Brand name**  *(Include weight and age information)* | | |
| --- | --- | --- | --- | --- | --- | --- | --- | --- | --- | --- | --- | --- | --- | --- |
|  |  | | | | |  |  |  |  |  |  |  |  |  |
| **5. Manufacturer** | | **6. Country of manufacture** | | | **7. Package size**  There are a total of  [___\|___\|___\|___] tablets/ suppositories/ granule packs in each:  1 = Package  2 = Pot/tin  [___] | | **8. Is product a fixed-dose combination (FDC)**  1 = Yes  0 = No  8 = Don’t   know  [___] | | | **9. Amount sold/distributed in the last 7 days to individual consumers** (*Record # of packages / tins described in* Q7 *OR record the total # of tablets / suppositories / granule packs sold*)  This outlet sold [___\|___\|___] **packages/ tins** in the last 7 days    **OR**  This outlet sold [___\|___\|___\|___] **tablets/ suppositories or granule packs** in the last 7 days  ***Not applicable = 9995; Refused = 9997; Don’t know = 9998*** | | | | **10. Stocked out at any point in the past 3 months?**  1 = Yes  0 = No  8 = Don’t   know  [___] |
| **11. Retail selling price**  [___\|___]  **tablets, suppositories or granule packs** cost an individual customer  [___\|___\|___\|___\|___]___] Dong | | | **12. Wholesale purchase price**  For the outlet’s most recent wholesale purchase  [___\|___\|___\|___]  **tablets, suppositories or granule packs** cost  [___\|___\|___\|___\|___\|___]___] Dong | | | | | | **13. Why do you stock this medicine [SHOW PRODUCT]?**  ***Do not read list***.  ***Circle ALL responses given***  Free supply A  Profitable B  Recommended by the government C  Low price D  Customer demand or preference E  Positive brand reputation F  Often prescribed by doctors G  Most effective for treating malaria H  Don’t know X  Other Z  ***specify*** [_________________________________] | | | | **14. Comments** | |
| ***Free = 00000***  ***Refused = 99997 Don’t know = 99998*** | | | | ***Free = 000000***  ***Refused = 999997***  ***Don’t know = 999998*** | | | | |  |  |  |  |  |  |

Tablet Audit Sheet [__|__] of [__|__]

|  | **1. Generic name** | | | | **2. Strength**  [__\|__\|__\|__].[__]mg/[__\|__\|__].[__] mL  [__\|__\|__\|__].[__]mg/[__\|__\|__].[__] mL  [__\|__\|__\|__].[__]mg/[__\|__\|__].[__] mL  **(*Note: no mL recorded for powder injection*)** | | | **2a. Is this base strength?**  [__]  1 = Yes  [__] 0 = No  8 = Don’t know  [__]  ***If no, specify salt:***  [____________________] | | **3. Dosage form**  1 = Syrup  2 = Suspension  3 = Liquid inj.  4 = Powder inj.  5 = Drops  6 = Other ***(specify)*** [___________]  [___] | |
| --- | --- | --- | --- | --- | --- | --- | --- | --- | --- | --- | --- |
|  |  | | | |  |  |  |  |  |  |  |
| **4. Brand name**  *(Include weight and age information)* | | | **5. Manufacturer** | **6. Country of manufacture** | | | **7. Package size**  There are a total of  [___\|___\|___\|___].[__] mL  (or mg for powder injections) in each:  1 = Bottle  2 = Ampoule/vial  [___] | **8. Amount sold/ distributed in the last 7 days to individual consumers**  This outlet sold  [___\|___\|___\|___] **bottles, ampoules or vials** in the  last 7 days  ***Refused = 9997;***  ***Don’t know = 9998*** | | | **9. Stocked out at any point in the past 3 months?**  1 = Yes  0 = No  8 = Don’t   know  [___] |
| **10. Retail selling price**  [___\|___\|___]  **bottles ampoules or vials** cost an individual customer  [___\|___\|___\|___\|___\|___] Dong | | **11. Wholesale purchase price**  For the outlet’s most recent wholesale purchase:  [___\|___\|___\|___]  **bottles, ampoules or vials**  cost  [___\|___\|___\|___\|___\|___]___\| Dong | | | | **12. Why do you stock this medicine [SHOW PRODUCT]?**  ***Do not read list***.  ***Circle ALL responses given***  Free supply A  Profitable B  Recommended by the government C  Low price D  Customer demand or preference E  Positive brand reputation F  Often prescribed by doctors G  Most effective for treating malaria H  Don’t know X  Other Z  ***specify*** [_________________________________] | | | **13. Comments** | | |
| ***Free = 00000***  ***Refused = 99997***  ***Don’t know = 99998*** | | ***Free = 000000***  ***Refused = 999997***  ***Don’t know = 999998*** | | | |  |  |  |  |  |  |

Non-Tablet Audit Sheet [___|___] of [___|___]

| A16. Are there any antimalarial medicines that are out of stock today, but that you stocked in the  past **3 months?**  1 = Yes ***go to A17***  0 = No ***go to Section 3: Diagnostic Audit***  8 = Don’t know ***go to Section 3: Diagnostic Audit*** | [___] |
| --- | --- |
| A17. Do you know the names of the treatments that are out of stock?  ***Will accept generic or brand names. Record one medicine per line.***  1 = Yes, ***specify***  [_______________________________________] [_______________________________________]  [_______________________________________] [_______________________________________]  [_______________________________________] [_______________________________________]  [_______________________________________] [_______________________________________]  [_______________________________________] [_______________________________________]  0 = No | [___] |

***Interviewer: Go to Section 3: Diagnostic Audit***

| **Section 3: Diagnostic Audit**  *This section is about availability of malaria blood testing. Completing the questions may require speaking with more than 1 staff member at the outlet. If the respondent does not know the answer to a question in this section, ask to speak with another staff member who can provide the information.* |
| --- |

| D1. Malaria rapid diagnostic tests, also called RDTs, are small, individually wrapped blood tests that are able to quickly diagnose whether a person has malaria. ***Show RDT images in prompt card***  Are malaria RDTs available here today?  1 = Yes ***Proceed to the RDT audit***  0 = No  ***go to D3***  Don’t know ***ask to speak with a respondent who has this information*** | [___] | |
| --- | --- | --- |
| D2. Please show us the full range of RDTs that you currently have in stock. | |  |

***Proceed to the RDT audit.***

***If additional audit sheets are used, add these sheets after the ones provided and staple the questionnaire again. All pages should be in order before you move onto the next outlet.***

***Number each RDT by assigning a Product Number.***

***Number each audit sheet used in the spaces provided at the bottom of the page.***

| **1. Brand name** | | **2. Antigen test**  *(circle ALL that apply)*  Not indicated **Z**  HRP2 **A**  pLDH **B**  Aldolase **C** | | | **3. Parasite species**  *(circle ALL that apply)*  Not indicated **Z**  Pf **A**  Pv **B**  Po **C**  pm **D**  pan **E**  vom/Pvom **F**  other **G**  Specify **[_____________________]** | | | **4. Manufacturer** | | | **5. Country of Manufacture** | **6. Lot Number** | | |
| --- | --- | --- | --- | --- | --- | --- | --- | --- | --- | --- | --- | --- | --- | --- |
| **6a. Is this RDT packaged as a**  **self-test kit? Show prompt card.**  1=Yes  0=No  8=Don’t know  [___] | | | **7. What is the name of your supply company for this product? Record the name of all suppliers.**  **[_______________________]**  ***Never placed order* = 95**  ***Refused* = 97**  ***Don't know* = 98** | | | **8. When did you first place an order for this product? Record month and year.**  [___\|___\| - \|__2_\|_0_\|___\|___] *Month - Year*  ***Never placed order* = 9995**  ***Refused* = 9997**  ***Don't know* = 9998** | | | | **9. What was the date of your most recent order for this product? Record month and year.**  [___\|___\| - \|_2_\|_0_\|___\|___]  *Month - Year*  ***Never placed order* = 9995**  ***Refused* = 9997**  ***Don't know* = 9998** | | | **10. How many RDT cassettes did you purchase for your most recent order ?**  [___\|___\|___\|___]  ***Never placed order* = 9995**  ***Refused = 9997***  ***Don’t know* = 9998** | |
| **11. Number of tests sold/ distributed /used in the last 7 days to individual consumers**  *(Record total # of tests)*  This outlet sold or distributed  [___\|___\|___\|___] **tests** in the last 7 days  ***Refused = 9997; Don’t know=9998*** | **12. Has this test been stocked out at any point in the past 3 months?**  1 = Yes  0 = No  8 = Don’t know  [___] | | | **13a. Do you or other staff use this brand of RDT to test clients here at this facility/outlet?**  1 = Yes  0 = No ***go to 16a***  8 = Don’t know ***go to 16a***  [___]    **13b. If yes, what is the total cost for an adult to have a test conducted with this RDT, including RDT cost and service fee?**  [___\|___\|___\|___\|___\|___] Dong  **13c. If yes, what is the total cost for a child under the age of five to have a test conducted with this RDT, including RDT cost and service fee?**  [___\|___\|___\|___\|___\|___] Dong | | | **14a. Does this facility/outlet provide this brand of RDT for clients to take away for testing somewhere else?**  1 = Yes  0 = No ***go to 17***  8 = Don’t know ***go to 17***  [___]    **14b. If yes, what is cost of this RDT for an adult?**  [___\|___\|___\|___\|___\|___] Dong  **14c. If yes, what is the cost of this RDT for a child under the age of five?**  [___\|___\|___\|___\|___\|___] Dong | | **15. Wholesale purchase price**  For the outlet’s most recent wholesale purchase:  [___\|___\|___\|___] **tests** cost  [___\|___\|___\|___\|___\| ___\|___]Dong  ***Free = 000000***  ***NA = 999995***  ***Refused = 999997***  ***Don’t know=999998*** | | | **16.**  **Why do you stock this RDT [SHOW RDT]?**  ***Do not read list***  ***Select all responses given***  A = Free supply  B = Profitable  C = Recommended by the government  D = Low price  E = Customer demand or preference  F = Positive brand reputation  X = Don’t know  Z = Other ***Specify***  [*_______________________*] | | **17. Comments** |
|  |  |  |  | ***Free = 00000; NA = 99995; Refused = 99997; Don’t know=99998*** | | | | |  |  |  |  |  |  |

RDT Audit Sheet [___|___] of [___|___]

**RDT stock outs**

| D3. Are there any malaria RDTs that are out of stock today, but that you stocked in the past **3 months**?  1 = Yes  0 = No ***go to D11***  8 = Don’t know ***go to D11*** | [___] |
| --- | --- |
| D4. Do you know the brand names of the malaria RDTs that are out of stock?  ***Record one brand per line.***  1 = Yes, ***specify***  [________________________________________________________________________]  [________________________________________________________________________]  [________________________________________________________________________]  0 = No | [___] |
